# Effective Use of Intravenous Lignocaine for Radiotherapy-Induced Brachial Plexopathy

**DOI:** 10.7759/cureus.68668

**Published:** 2024-09-04

**Authors:** Mak Wen Jie, Ying Mao Gn, Tham Kar Mun

**Affiliations:** 1 Anaesthesiology, Sengkang General Hospital, Singapore, SGP; 2 Anaesthesiology, Singapore General Hospital, Singapore, SGP; 3 Pain Medicine, Singapore General Hospital, Singapore, SGP

**Keywords:** chronic pain, treatment complication, breast cancer, lignocaine infusion, radiation-induced brachial plexopathy

## Abstract

Radiotherapy-induced brachial plexopathy (RIBP) is a rare but debilitating complication of breast cancer treatment. There is limited information available on the effective treatments for this condition. We present the case of a 68-year-old female with well-controlled schizophrenia and a history of breast cancer who was referred to our pain management clinic for dysesthesia in the left upper limb secondary to RIBP. The patient exhibited a remarkable response to intravenous (IV) lidocaine infusion, with near-complete resolution of her symptoms. This case highlights the potential of IV lidocaine infusion as a valuable component of a multimodal strategy for managing RIBP.

## Introduction

Radiation-induced brachial plexopathy (RIBP) is a rare complication arising from radiotherapy used as an adjuvant treatment for cancers in the neck, axilla, and thorax. It occurs because of the proximity of the brachial plexus to the target radiation site, resulting in its inadvertent exposure to radiation. The incidence of RIBP in breast cancer was reported to be 1.8% [1]. As the diagnosis is rare, there is a paucity of literature on the successful management of RIBP. In this case report, we present a case of a patient diagnosed with RIBP who demonstrated an excellent response to intravenous (IV) lidocaine infusion. To our knowledge, this is the first report whereby IV lidocaine infusion has been used to successfully manage the symptoms of RIBP.

## Case presentation

A 68-year-old female with a history of well-controlled schizophrenia and cervical radiculopathy was diagnosed with left breast cancer with metastases to the axillary lymph nodes in 2017. She underwent a left simple mastectomy with axillary clearance and received chemoradiotherapy in the same year, which included paclitaxel, adriamycin, and 40 grays (Gy) of radiotherapy. After completing her breast cancer treatment, the patient reported severe left upper limb paresthesia years later, which was distinct from her previous radicular symptom.

**Figure 1:**
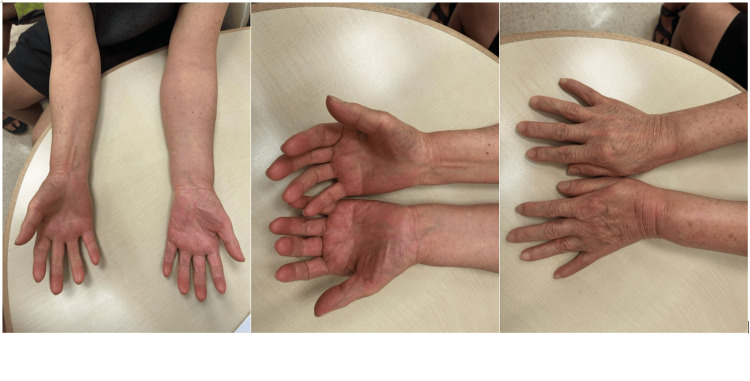
Images showing the patient's affected left upper limb

The patient was first diagnosed with cervical radiculopathy in 2017 when she complained of numbness over the left fingers and forearm. Her MRI of the cervical spine in 2017 showed multilevel degenerative disc disease and cervical spondylosis with exit foraminal stenosis at different levels. Her symptom was controlled with gabapentin 300 mg once daily. However, in 2024, she was referred to a pain management center for worsening dysesthesia over her left upper limb. She described the pain as a "constant electric shock sensation traveling along the entire left upper limb." The pain was severe, significantly impacting her mood and daily activities. Examination revealed swelling, redness, and wasting of the small muscles in the left upper limb, along with weakness (Figure 1). 

**Figure 2:**
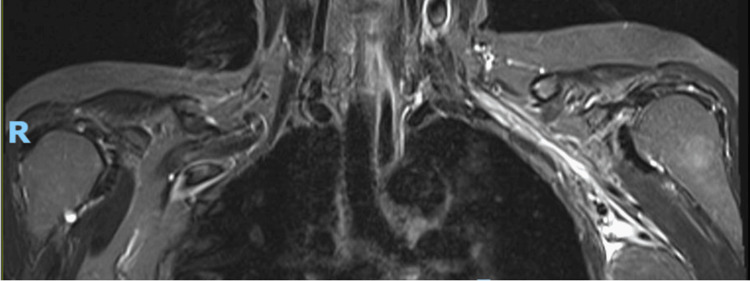
MRI brachial plexus: coronal fat-suppressed T2-weighted image showed abnormal thickening, T2 hyperintensity, and enhancement involving the left-sided trunks, divisions, and cords of the brachial plexus

MRI of the left brachial plexus showed thickening, T2W hyperintensity, and enhancement involving the trunks, divisions, and cords, favoring the diagnosis of RIBP (Figure 2). The nerve conduction study (NCS) reported reduced sensory amplitudes, suggesting ongoing plexopathy (Table 1).

**Table 1:** Report of the nerve conduction study of the patient

Nerve	Onset Latency ms	Amplitude microV	Conduction Velocity m/s
Medianus Sensory Left			
Wrist: Digit II	2.79	22.8	46.6
Medianus Sensory Right			
Wrist: Digit II	2.89	48.0	45.0
Ulnaris Sensory Left			
Wrist: Digit V	Not Recordable	Not Recordable	Not Recordable
Ulnaris Sensory Right			
Wrist: Digit V	2.18	28.7	50.5
Cutaneous anterior Branch Lateral Sensory Left			
Lateral Biceps Tendon: Between Flexor Carpi Radialis Radial Styloid	1.96	9.8	61.2
Cutaneous Anterior Branch Lateral Sensory Right			
Lateral Biceps Tendon: Between Flexor Carpi Radialis Radial Styloid	1.98	23.3	60.6
Cutaneous Anterior Branch Medial Sensory Left			
Medial Median Nerve: Anteromedial Forearm	Not Recordable	Not Recordable	Not Recordable
Cutaneous Anterior Branch Medial Sensory Right			
Medial Median Nerve: Anteromedial Forearm	2.23	6.1	53.8
Radialis Sensory Left			
Wrist: Extensor Pollicis Longus Tendon	1.96	12.8	51
Radialis Sensory Right			
Wrist: Extensor Pollicis Longus Tendon	1.90	19.2	52.6

Despite escalating her gabapentin dose to 300 mg ter die sumendum (TDS), converting to pregabalin 75 mg omni nocte (ON), and undergoing physiotherapy, her symptoms showed minimal improvement. Other neuropathic agents were limited because of her psychiatric history and use of multiple antipsychotics. Consequently, she was started on a trial of IV lidocaine infusion. She received a bolus of 1 mg/kg of 1% lidocaine followed by 1 mg/kg/h of 0.5% lidocaine for four hours over five consecutive days. She reported a significant reduction in her pain score on the numeric rating scale (0: no pain; 10: severe pain), improving from 10/10 to 3/10 after five cycles of lidocaine infusion, with no side effects. A follow-up clinic visit two months post-intervention showed persistent significant improvement in pain relief.

## Discussion

RIBP is a rare but severe complication following breast cancer treatment. Its incidence ranges from 1.2% to 1.8% and typically occurs six months to 20 years post-radiotherapy (median: four years) [1-3]. As there may be significant latent period before the onset of symptoms, RIBP diagnosis may be missed. Clinicians must consider this diagnosis in patients presenting with relevant neurological symptoms.

The exact mechanism of RIBP is not well understood, but it is thought to involve radiotherapy-induced fibrosis of neural and perineural soft tissues, damaging axons, and Schwann cells. The long latency onset suggests that RIBP may result from decreased nerve repair capacity to mild recurrent trauma from normal activity over the years, aligning with the "double crush" phenomenon. Risk factors include concurrent chemotherapy and radiotherapy doses exceeding 55 Gy [4].

Symptoms of RIBP are nonspecific, including numbness, paresthesia, dysesthesia, lymphedema, and motor weakness, requiring a high index of suspicion for diagnosis [3]. Severe cases may involve shoulder joint fibrosis, limiting the range of motion. Motor weakness occurs because of axonal damage from fibrosis and ischemia and has been reported to be irreversible once it is established [2]. While MRI usually shows abnormal isointense or hypointense signals of the brachial plexus relative to muscle on T2-weighted images, hyperintense signals were also reported. Electromyography (EMG) is another valid means to diagnose RIBP; myokymia on the EMG is an important finding of RIBP [5].

In our case, the diagnosis of RIBP was challenging because of preexisting paresthesia from cervical spondylosis, leading the oncological team to initially discount RIBP. Detailed history subsequently revealed changes in symptoms over the years. Differential diagnoses included worsening cervical spondylosis, brachial plexus tumor invasion, axillary clearance injury, or paraneoplastic syndrome. Clinical features of complex regional pain syndrome (CRPS) were also noted [6]. The diagnosis of RIBP was confirmed by MRI, and CRPS was excluded as the patient did not meet the diagnostic criteria [2,4,6].

There is currently no consensus on the treatment for RIBP. A multimodal approach is generally adopted by pain physicians for the management of RIBP, as for other complex pain syndromes. Neuropathic agents are often used, although there is currently no literature to support their use in RIBP. Our patient did not display any improvement in symptoms with gabapentinoids. Physiotherapy is also useful to preserve muscle strength, maintain range of motion in the affected joint, and limit lymphedema. Other treatment modalities include epidural injection of steroids and local anesthetic agents, brachial plexus blocks, and spinal cord stimulation [2].

IV lidocaine has been a cornerstone in the management of neuropathic pain. It is postulated that lidocaine inhibits overexpressed voltage-gated sodium channels on cell membranes of injured peripheral nerves, dorsal root ganglia, and adjacent neurons, thereby silencing ectopic discharges, suppressing inflammatory processes, and modulating both inhibitory and excitatory neurotransmission [7,8]. A systematic review and meta-analysis concluded that systemic local anesthetics, including IV lidocaine, are superior to placebo and equal to other pain modulators for relieving neuropathic pain [9]. Lidocaine has been successfully used in similar contexts of brachial plexus pain including the treatment of complex regional pain syndrome and cancer pain [6,10]. While the successful use of IV lidocaine infusion to treat pain from traumatic brachial plexus injury has been reported where the patient's pain was largely resolved, to our knowledge, this is the first report of RIBP demonstrating remarkable response to IV lidocaine infusion [11]. Our patient achieved near resolution of her pain and dysesthesias with a course of lidocaine infusion.

## Conclusions

RIBP is rare and can cause severe intractable pain and significantly worsen the quality of life. As the lifespan of cancer survivors increases with improved oncological treatment, the incidence of RIBP is expected to rise. Our patient with RIBP after axillary radiotherapy was successfully managed with IV lignocaine infusion. This highlights its valuable role as part of a multimodal strategy for managing complex pain syndromes. This may allude to the role of sodium channel upregulation as a key mediator in radiotherapy-induced nerve injuries: future studies in this area will be invaluable to aid clinicians in determining the optimal neuropathic agent to treat RIBP.

## References

[1] Gosk J, Rutowski R, Reichert P, Rabczyński J (2007). Review article: radiation-induced brachial plexus neuropathy - aetiopathogenesis, risk factors, differential diagnostics, symptoms and treatment. Folia Neuropathol.

[2] Warade AC, Jha AK, Pattankar S, Desai K (2019). Radiation-induced brachial plexus neuropathy: a review. Neurol India.

[3] de Oliveira AJ, Castro JP, Foroni LH, Siqueira MG, Martins RS (2020). Treatment of radiation-induced brachial plexopathy with omentoplasty. Autops Case Rep.

[4] Schierle C, Winograd JM (2004). Radiation-induced brachial plexopathy: review. Complication without a cure. J Reconstr Microsurg.

[5] Gu B, Yang Z, Huang S (2014). Radiation-induced brachial plexus injury after radiotherapy for nasopharyngeal carcinoma. Jpn J Clin Oncol.

[6] Harden RN, McCabe CS, Goebel A, Massey M, Suvar T, Grieve S, Bruehl S (2022). Complex regional pain syndrome: practical diagnostic and treatment guidelines, 5th edition. Pain Med.

[7] Doo AR, Shin YS, Yoo S, Park JK (2018). Radiation-induced neuropathic pain successfully treated with systemic lidocaine administration. J Pain Res.

[8] Hermanns H, Hollmann MW, Stevens MF, Lirk P, Brandenburger T, Piegeler T, Werdehausen R (2019). Molecular mechanisms of action of systemic lidocaine in acute and chronic pain: a narrative review. Br J Anaesth.

[9] Tremont-Lukats IW, Challapalli V, McNicol ED, Lau J, Carr DB (2005). Systemic administration of local anesthetics to relieve neuropathic pain: a systematic review and meta-analysis. Anesth Analg.

[10] Stowe HB, Mullins BT, Chera BS (2020). Hyperbaric oxygen therapy for radiation-induced brachial plexopathy, a case report and literature review. Rep Pract Oncol Radiother.

[11] Hawley P, Ridley J, Youngson M (2021). Dramatic response to lidocaine infusion for pain from brachial plexus avulsion injury. Pain Med Case Rep.

